# Supplementary material for: Genetically Predicted Levels of Serum Metabolites and Risk of Sarcopenia: A Mendelian Randomization Study
Source: Nutrients. 2023 Sep 13;15(18):3964. doi: 10.3390/nu15183964 (PMC10536442; doi:10.3390/nu15183964)
Supplement: Supplementary file 1 [file nutrients-15-03964-s001.zip › nutrients-2577942-supplementary.pdf]

Table of Contents

Supplementary Table 1. Detailed information on GWAS summary statistics or individual-level genetic data used in our analysis.. 2

Supplementary Table 2. Detailed information on selected single nucleotide polymorphisms robustly and independently related to serum metabolites used in discovery GWAS.. 3

Supplementary Table 3. Sensitive results of the associations between serum metabolites and the risk of sarcopenia as well as the global test of MR-PRESSO 14

Supplementary Table 4. Sensitive results of the associations between serum metabolites and appendicular lean mass as well as the global test of MR-PRESSO........................... 17

Supplementary Table 5. Sensitive results of the associations between serum metabolites and grip strength as well as the global test of MR-PRESSO.. 20

Supplementary Table 6. Detailed information on selected single nucleotide polymorphisms robustly and independently related to sarcopenic indices...................... 22

Supplementary Table 7. Results of the reverse associations between sarcopenic indices and identified metabolites.. 23

Supplementary Figure 1. Suggestive significant association between serum metabolites with sarcopenia. 27

Table S1. Detailed information on GWAS summary statistics or individual-level genetic data used in our analysis.

Traits	Sample size	SNP	Dataset reference
Serum metabolites	7,824	2.1 million SNPs	PMID: 24816252
Sarcopenia	324,976	10,894,596	UK biobank
Appendicular lean mass	324,976	10,894,596	UK biobank
Hand grip strength	324,976	10,894,596	UK biobank

Table S2. Detailed information on selected single nucleotide polymorphisms robustly and independently related to serum metabolites used in discovery GWAS.

Trait	Subcategory	N SNP	Variance	Minimum F statistic
Glutamine	Amino acid	8	4.3%	20.250
Tryptophan	Amino acid	193	65.8%	18.948
Histidine	Amino acid	7	5.5%	19.724
Leucine	Amino acid	208	68.7%	17.640
Cholesterol	Lipid	47	18.3%	18.439
Phenylalanine	Amino acid	5	3.7%	20.055
Creatinine	Amino acid	26	10.9%	19.083
Lactate	Carbohydrate	13	5.7%	19.507
3-hydroxybutyrate (BHBA)	Lipid	10	5.8%	19.881
Arabinose	Carbohydrate	5	7.1%	19.885
Mannose	Carbohydrate	21	13.9%	19.464
Pyruvate	Carbohydrate	14	5.1%	19.637
Uridine	Nucleotide	21	8.2%	19.866
Linoleate (18:2n6)	Fatty acid	17	5.1%	19.714
Allantoin	Nucleotide	19	12.6%	19.314
Arachidonate (20:4n6)	Fatty acid	21	12.8%	19.753
Deoxycholate	Lipid	17	10.2%	19.677
Margarate (17:0)	Fatty acid	8	3.8%	20.360
Inosine	Nucleotide	11	12.4%	19.849
Isoleucine	Amino acid	18	8.0%	18.778
Threonine	Amino acid	20	8.4%	19.010
Tyrosine	Amino acid	34	14.2%	18.778
Lysine	Amino acid	25	9.1%	19.753

Methionine	Amino acid	22	7.4%	18.598
Malate	Energy	17	6.6%	19.674
Palmitate (16:0)	Fatty acid	28	9.7%	19.472
Nonadecanoate (19:0)	Fatty acid	16	8.3%	19.951
Stearate (18:0)	Fatty acid	43	16.7%	19.753
Oleate (18:1n9)	Fatty acid	20	6.1%	19.360
Pentadecanoate (15:0)	Fatty acid	20	7.3%	19.830
Myristate (14:0)	Fatty acid	23	9.5%	19.564
Pipecolate	Amino acid	16	8.4%	19.193
Ornithine	Amino acid	11	4.3%	19.360
5-oxoproline	Amino acid	23	12.9%	19.440
Pantothenate	Cofactors and vitamins	26	10.9%	18.778
4-acetamidobutanoate	Amino acid	35	16.3%	19.262
Alpha-tocopherol	Cofactors and vitamins	11	6.8%	19.360
Citrate	Energy	46	15.8%	18.626
Glycerate	Carbohydrate	17	5.2%	19.564
Guanosine	Nucleotide	13	13.0%	19.686
N-acetylalanine	Amino acid	23	6.9%	19.262
Urate	Nucleotide	22	13.0%	18.778
Ursodeoxycholate	Lipid	11	11.5%	19.700
Arginine	Amino acid	22	10.3%	19.284
Ascorbate (Vitamin C)	Cofactors and vitamins	14	21.7%	19.737
Heptanoate (7:0)	Fatty acid	36	12.8%	19.083
Laurate (12:0)	Fatty acid	41	16.0%	19.262
Valine	Amino acid	6	4.1%	21.007
Urea	Amino acid	14	5.0%	19.564

Cortisol	Lipid	13	7.7%	19.041
Cortisone	Lipid	40	14.9%	18.778
Proline	Amino acid	38	21.0%	19.262
Citrulline	Amino acid	47	16.3%	18.778
Biliverdin	Cofactors and vitamins	19	26.1%	19.807
Serotonin (5HT)	Amino acid	16	8.6%	19.307
Gamma-glutamylglutamine	Peptide	22	9.9%	19.440
Gamma-glutamyltyrosine	Peptide	47	17.3%	19.262
Hypoxanthine	Nucleotide	25	11.2%	19.360
Betaine	Amino acid	25	13.4%	19.184
Xanthine	Nucleotide	5	2.8%	19.951
Phosphate	Energy	5	4.2%	20.250
3-methoxytyrosine	Amino acid	16	8.4%	19.100
Pelargonate (9:0)	Fatty acid	36	12.5%	18.778
Undecanoate (11:0)	Fatty acid	28	12.2%	18.778
Beta-hydroxyisovalerate	Amino acid	23	9.9%	19.100
Taurodeoxycholate	Lipid	13	18.9%	19.580
Glycerol	Lipid	18	7.4%	19.100
Kynurenine	Amino acid	43	19.7%	18.778
Mannitol	Carbohydrate	13	8.1%	19.966
Glycerol 3-phosphate (G3P)	Lipid	14	5.0%	19.205
Acetylphosphate	Energy	18	5.8%	18.948
Carnitine	Lipid	226	82.3%	19.262
Choline	Lipid	25	7.8%	19.141
N-acetylornithine	Amino acid	19	30.5%	19.319
N1-methyladenosine	Nucleotide	8	3.5%	19.225

3-methyl-2-oxovalerate	Amino acid	34	14.5%	19.262
3-methylhistidine	Amino acid	10	6.5%	19.597
3-phenylpropionate (hydrocinnamate)	Amino acid	16	10.1%	19.589
Phenylacetate	Amino acid	9	8.5%	19.551
Glycerophosphorylcholine (GPC)	Lipid	19	9.4%	19.109
Aspartate	Amino acid	4	2.1%	19.857
X-04499--3,4-dihydroxybutyrate	Amino acid	21	7.5%	19.010
Dihomo-linoleate (20:2n6)	Fatty acid	9	3.4%	19.536
2-hydroxystearate	Lipid	36	11.1%	19.083
Indolelactate	Amino acid	19	8.6%	19.425
Glycylvaline	Peptide	8	9.4%	19.732
Gamma-glutamylleucine	Peptide	37	14.1%	19.418
Eicosapentaenoate (EPA; 20:5n3)	Fatty acid	12	7.0%	19.843
Estrone 3-sulfate	Lipid	13	67.1%	19.553
Glycocholate	Lipid	9	7.7%	20.086
Glycodeoxycholate	Lipid	7	12.5%	20.491
Taurochenodeoxycholate	Lipid	13	9.9%	19.475
Taurocholate	Lipid	16	21.4%	19.856
Docosahexaenoate (DHA; 22:6n3)	Fatty acid	6	4.2%	19.959
1-stearoylglycerophosphoinositol	Lipid	7	7.1%	19.843
Myo-inositol	Lipid	40	18.6%	18.778
Glucose	Carbohydrate	38	15.4%	18.948
1,5-anhydroglucitol (1,5-AG)	Carbohydrate	31	15.0%	19.100
2-hydroxybutyrate (AHB)	Amino acid	18	7.3%	19.271
3-methyl-2-oxobutyrate	Amino acid	18	6.5%	19.284
1,6-anhydroglucose	Carbohydrate	15	26.7%	19.688

1-palmitoylglycerol (1-monopalmitin)	Lipid	14	5.5%	20.084
1-oleoylglycerol (1-monoolein)	Lipid	16	7.5%	20.022
1-stearoylglycerol (1-monostearin)	Lipid	25	11.0%	19.425
2-hydroxyisobutyrate	Amino acid	19	7.9%	19.564
4-methyl-2-oxopentanoate	Amino acid	14	6.3%	19.905
Phenyllactate (PLA)	Amino acid	19	11.3%	19.612
Homocitrulline	Amino acid	7	5.9%	19.612
Aspartylphenylalanine	Peptide	7	6.1%	19.786
Levulinate (4-oxovalerate)	Amino acid	61	22.7%	18.490
Palmitoylcarnitine	Lipid	10	4.0%	19.205
Cholate	Lipid	8	5.5%	19.831
1-linoleoylglycerol (1-monolinolein)	Lipid	15	13.4%	19.587
Indoleacetate	Amino acid	22	8.8%	19.297
Hyodeoxycholate	Lipid	17	12.5%	20.060
3-indoxyl sulfate	Amino acid	5	5.2%	20.312
N-acetylglycine	Amino acid	16	12.2%	19.440
Bilirubin (Z,Z)	Cofactors and vitamins	11	16.0%	19.540
Creatine	Amino acid	8	6.1%	19.795
Erythrose	Carbohydrate	8	3.5%	19.546
Threonate	Cofactors and vitamins	18	9.7%	19.499
Cysteine	Amino acid	19	9.2%	19.585
Pyroglutamylglycine	Peptide	4	7.9%	19.803
N-(2-furoyl)glycine	Xenobiotics	26	12.4%	19.548
DSGEGDFXAEGGGVR	Peptide	13	7.4%	19.890
Pyridoxate	Cofactors and vitamins	12	7.1%	19.581
Androsterone sulfate	Lipid	22	20.7%	19.527

3-carboxy-4-methyl-5-propyl-2-furanpropanoate (CMPF)	Lipid	14	7.3%	19.905
3-(4-hydroxyphenyl)lactate	Amino acid	23	9.8%	19.721
Acetylcarnitine	Lipid	21	10.3%	19.100
Serine	Lipid	35	13.1%	18.778
Trans-4-hydroxyproline	Amino acid	6	4.3%	18.934
Glutamate	Amino acid	3	1.0%	20.379
Hexanoylcarnitine	Lipid	19	13.0%	19.711
Glycine	Amino acid	26	18.7%	19.010
Alanine	Amino acid	39	13.4%	19.262
Glycochenodeoxycholate	Lipid	14	8.0%	19.531
2-aminobutyrate	Amino acid	46	14.9%	18.778
Scyllo-inositol	Lipid	12	6.4%	20.043
Dodecanedioate	Lipid	6	8.3%	21.319
Gamma-glutamylvaline	Peptide	15	5.5%	19.691
Indolepropionate	Amino acid	17	9.3%	19.314
Butyrylcarnitine	Lipid	33	32.4%	19.566
Myristoleate (14:1n5)	Fatty acid	15	8.6%	19.141
Dehydroisoandrosterone sulfate (DHEA-S)	Lipid	22	7.4%	19.360
Propionylcarnitine	Lipid	35	16.9%	19.546
Caproate (6:0)	Fatty acid	37	12.9%	19.262
Caprylate (8:0)	Fatty acid	45	16.3%	18.778
10-undecenoate (11:1n1)	Fatty acid	29	15.6%	19.255
Docosapentaenoate (n3 DPA; 22:5n3)	Fatty acid	11	7.3%	19.334
Phenol sulfate	Amino acid	16	6.5%	19.699
Bilirubin (E,E)	Cofactors and vitamins	10	10.3%	19.787
Heme	Cofactors and vitamins	13	5.5%	19.542

1-linoleoylglycerophosphoethanolamine	Lipid	13	5.9%	19.612
3-dehydrocarnitine	Lipid	27	11.6%	19.425
Pyroglutamine	Amino acid	18	10.8%	19.297
C-glycosyltryptophan	Amino acid	23	11.3%	19.464
X-03056--N-[3-(2-Oxopyrrolidin-1-yl)propyl]acetamide	Amino acid	30	15.9%	19.425
X-11422--xanthine	Nucleotide	8	4.7%	19.905
X-11423--O-sulfo-L-tyrosine	Amino acid	52	19.5%	18.778
X-11445--5-alpha-pregnan-3beta,20alpha-disulfate	Lipid	15	15.3%	19.598
HWESASXX	Peptide	6	3.9%	21.674
X-11593--O-methylascorbate	Cofactors and vitamins	49	28.2%	18.778
Adrenate (22:4n6)	Fatty acid	11	6.9%	19.819
Homostachydrine	Xenobiotics	6	8.1%	20.250
ADSGEGDFXAEGGGVR	Peptide	10	6.7%	19.546
X-11786--methylcysteine	Amino acid	12	11.5%	19.360
X-11793--oxidized bilirubin	Cofactors and vitamins	15	11.2%	19.705
1-arachidonoylglycerophosphocholine	Lipid	21	13.4%	18.878
1-palmitoleoylglycerophosphocholine	Lipid	14	4.2%	18.985
Gamma-glutamylmethionine	Peptide	9	10.8%	19.930
Gamma-glutamylthreonine	Peptide	10	7.9%	19.787
Gamma-tocopherol	Cofactors and vitamins	13	9.8%	19.524
Gamma-glutamylphenylalanine	Peptide	38	12.7%	18.778
Isobutyrylcarnitine	Amino acid	12	8.9%	20.783
Pseudouridine	Nucleotide	28	9.5%	19.262
Valerate	Lipid	10	6.0%	19.701
Palmitoleate (16:1n7)	Fatty acid	10	5.0%	19.360
Alpha-ketoglutarate	Energy	18	9.2%	19.464

Erythronate	Carbohydrate	48	16.3%	18.985
Lathosterol	Lipid	18	16.5%	19.499
X-12095--N1-methyl-3-pyridone-4-carboxamide	Nucleotide	24	12.1%	19.677
X-12100--hydroxytryptophan	Amino acid	46	15.6%	18.985
Eicosenoate (20:1n9 or 11)	Fatty acid	13	4.7%	19.550
X-12244--N-acetylcarnosine	Peptide	24	13.8%	19.141
ADpSGEGDFXAEGGGVR	Peptide	7	6.6%	19.657
1-eicosatrienoylglycerophosphocholine	Lipid	20	9.4%	19.360
1-docosahexaenoylglycerophosphocholine	Lipid	8	3.2%	19.961
1-eicosadienoylglycerophosphocholine	Lipid	13	8.2%	19.592
X-12441--12-hydroxyeicosatetraenoate (12-HETE)	Lipid	14	14.2%	19.440
X-12442--5,8-tetradecadienoate	Lipid	12	8.2%	19.524
Octanoylcarnitine	Lipid	18	11.4%	19.699
Alpha-hydroxyisovalerate	Amino acid	15	9.1%	20.250
N-acetylthreonine	Amino acid	13	6.6%	19.314
Decanoylcarnitine	Lipid	15	9.9%	19.454
1-palmitoylglycerophosphocholine	Lipid	34	11.2%	19.425
1-heptadecanoylglycerophosphocholine	Lipid	10	3.9%	19.496
1-oleoylglycerophosphocholine	Lipid	17	5.9%	19.718
1-stearoylglycerophosphocholine	Lipid	13	6.3%	19.705
5-dodecenoate (12:1n7)	Fatty acid	19	10.0%	19.730
Stearidonate (18:4n3)	Fatty acid	11	5.5%	19.559
10-heptadecenoate (17:1n7)	Fatty acid	6	2.5%	19.774
10-nonadecenoate (19:1n9)	Fatty acid	7	3.9%	19.724
Epiandrosterone sulfate	Lipid	13	12.3%	19.612
Linolenate [alpha or gamma; (18:3n3 or 6)]	Fatty acid	4	2.9%	19.524

X-12510--2-aminooctanoic acid	Amino acid	16	15.6%	19.440
Bilirubin (E,Z or Z,E)	Cofactors and vitamins	16	14.3%	19.637
1-arachidonoylglycerophosphoinositol	Lipid	21	11.4%	19.843
Asparagine	Amino acid	42	19.4%	19.141
Stachydrine	Xenobiotics	7	5.1%	19.710
Isovalerylcarnitine	Amino acid	21	13.5%	20.084
Stearoylcarnitine	Lipid	6	2.2%	19.464
1-stearoylglycerophosphoethanolamine	Lipid	14	8.9%	19.507
1-linoleoylglycerophosphocholine	Lipid	15	7.3%	19.518
Bradykinin, des-arg(9)	Peptide	22	19.9%	19.701
Gamma-glutamylisoleucine	Peptide	26	13.9%	19.297
Laurylcarnitine	Lipid	13	6.7%	19.360
X-12990--docosapentaenoic acid (n6-DPA)	Fatty acid	12	13.1%	19.432
Isovalerate	Lipid	4	2.1%	19.753
X-13183--stearamide	Lipid	11	13.0%	19.420
7-methylguanine	Nucleotide	11	10.2%	19.193
Phenylacetylglutamine	Amino acid	18	7.5%	19.641
Pro-hydroxy-pro	Peptide	19	8.7%	18.889
N2,N2-dimethylguanosine	Nucleotide	61	32.4%	19.450
Cysteine-glutathione disulfide	Amino acid	9	11.7%	20.598
Oleoylcarnitine	Lipid	12	4.6%	19.630
1-arachidonoylglycerophosphoethanolamine	Lipid	26	11.7%	19.425
X-13431--nonanoylcarnitine	Lipid	16	15.7%	19.730
2-palmitoylglycerophosphocholine	Lipid	24	7.9%	20.084
2-oleoylglycerophosphocholine	Lipid	18	5.6%	19.507
2-stearoylglycerophosphocholine	Lipid	13	7.7%	19.531

2-linoleoylglycerophosphocholine	Lipid	21	7.1%	19.193
1-palmitoylglycerophosphoinositol	Lipid	11	8.3%	19.165
2-methylbutyroylcarnitine	Amino acid	26	8.3%	19.170
Hydroxyisovaleroyl carnitine	Amino acid	8	6.4%	19.774
Glutaroyl carnitine	Amino acid	35	23.6%	19.100
2-tetradecenoyl carnitine	Lipid	19	11.0%	19.720
4-hydroxyhippurate	Xenobiotics	8	12.8%	19.360
1-myristoylglycerophosphocholine	Lipid	7	4.7%	19.408
1-oleoylglycerophosphoethanolamine	Lipid	10	3.1%	19.440
1-palmitoylglycerophosphoethanolamine	Lipid	28	13.1%	18.778
3-(3-hydroxyphenyl)propionate	Amino acid	11	33.3%	19.874
Tetradecanedioate	Lipid	20	19.4%	19.765
2-hydroxypalmitate	Lipid	48	18.3%	18.626
Hexadecanedioate	Lipid	25	15.0%	19.753
Dihomo-linolenate (20:3n3 or n6)	Fatty acid	23	9.8%	19.225
Threitol	Carbohydrate	13	6.8%	19.408
P-cresol sulfate	Amino acid	15	8.3%	19.623
X-14189--leucylalanine	Peptide	13	16.1%	20.174
X-14205--alpha-glutamyltyrosine	Peptide	18	25.6%	19.670
X-14208--phenylalanylserine	Peptide	14	17.3%	19.518
X-14304--leucylalanine	Peptide	21	22.1%	19.803
X-14450--phenylalanylleucine	Peptide	5	5.1%	20.372
Gamma-glutamylglutamate	Peptide	10	30.2%	20.188
Octadecanedioate	Lipid	10	6.3%	19.701
Leucylleucine	Peptide	11	8.7%	19.641
7-alpha-hydroxy-3-oxo-4-cholestenoate (7-Hoca)	Lipid	16	4.8%	19.464

N-Butyl Oleate	Lipid	9	4.3%	19.843
Dimethylarginine (SDMA + ADMA)	Amino acid	35	15.1%	19.262
Taurolithocholate 3-sulfate	Lipid	11	6.7%	20.001
X-14977--vanillin	Xenobiotics	9	10.4%	19.905
Succinylcarnitine	Energy	44	22.9%	18.626
Tryptophan betaine	Amino acid	13	9.7%	19.645
Cyclo(leu-pro)	Peptide	15	11.8%	19.332
Chiro-inositol	Lipid	13	26.0%	19.549
5α-Adiol disulfate	Lipid	18	12.2%	19.649
4α-Adiol disulfate 1	Lipid	22	14.5%	19.665
4α-Adiol disulfate 2	Lipid	19	6.9%	19.464
2-hydroxyglutarate	Lipid	13	8.1%	19.447
Palmitoyl sphingomyelin	Lipid	48	15.0%	18.778
Phenylalanylphenylalanine	Peptide	4	2.8%	20.617
Cis-4-decenoyl carnitine	Lipid	10	7.0%	19.170
15-methylpalmitate (isobar with 2-methylpalmitate)	Lipid	14	7.3%	19.612

We only presented the information for the known serum metabolites if the number of SNPs in the extracted instrumental variables was more than 2.

Table S3. Sensitive results of the associations between serum metabolites and the risk of sarcopenia as well as the global test of MR-PRESSO.

Metabolites	Method	N SNP	Beta	se	P	OR	OR_lci95	OR_uci95	P global test
4-acetamidobutanoate	Inverse variance weighted	35	-2.34	1.10	0.0340	0.10	0.01	0.84	-
4-acetamidobutanoate	Weighted median	35	-1.24	1.74	0.4750	0.29	0.01	8.69	-
4-acetamidobutanoate	MR-PRESSO	35	-2.24	1.08	0.0460	0.11	0.01	0.89	>0.05
Biliverdin	Inverse variance weighted	19	0.91	0.43	0.0360	2.49	1.06	5.82	-
Biliverdin	Weighted median	19	0.46	0.52	0.3810	1.58	0.57	4.40	-
Biliverdin	MR-PRESSO	19	0.93	0.42	0.0400	2.54	1.11	5.82	>0.05
Taurocholate	Inverse variance weighted	16	0.42	0.20	0.0370	1.52	1.03	2.24	-
Taurocholate	Weighted median	16	0.43	0.28	0.1270	1.53	0.89	2.64	-
Taurocholate	MR-PRESSO	16	0.34	0.21	0.1310	1.40	0.92	2.12	>0.05
Myo-inositol	Inverse variance weighted	40	-1.85	0.91	0.0420	0.16	0.03	0.94	-
Myo-inositol	Weighted median	40	-1.69	1.30	0.1950	0.18	0.01	2.38	-
Myo-inositol	MR-PRESSO	40	-2.28	0.93	0.0180	0.10	0.02	0.63	>0.05
Phenyllactate	Inverse variance weighted	19	-2.07	0.95	0.0290	0.13	0.02	0.81	-
Phenyllactate	Weighted median	19	-2.72	1.41	0.0540	0.07	0.00	1.05	-
Phenyllactate	MR-PRESSO	19	-2.07	0.90	0.0340	0.13	0.02	0.74	>0.05
Levulinate (4-oxovalerate)	Inverse variance weighted	61	1.70	0.81	0.0350	5.50	1.12	26.89	-

Levulinate (4-oxovalerate)	Weighted median	61	2.71	1.29	0.0350	15.06	1.21	187.44	-
Levulinate (4-oxovalerate)	MR-PRESSO	61	1.39	0.72	0.0560	4.03	0.99	16.38	>0.05
Acetylcarnitine	Inverse variance weighted	21	2.39	1.00	0.0160	10.93	1.55	76.82	-
Acetylcarnitine	Weighted median	21	2.46	1.38	0.0740	11.76	0.79	175.60	-
Acetylcarnitine	MR-PRESSO	21	2.39	1.00	0.0260	10.92	1.55	76.80	>0.05
Docosapentaenoate	Inverse variance weighted	11	-1.85	0.85	0.0300	0.16	0.03	0.83	-
Docosapentaenoate	Weighted median	11	-0.42	1.12	0.7090	0.66	0.07	5.94	-
Docosapentaenoate	MR-PRESSO	11	-1.65	0.85	0.0780	0.19	0.04	1.01	>0.05
5-alpha-pregnan-3beta,20alpha-disulfate	Inverse variance weighted	15	-0.86	0.37	0.0190	0.42	0.20	0.87	-
5-alpha-pregnan-3beta,20alpha-disulfate	Weighted median	15	-0.97	0.48	0.0410	0.38	0.15	0.96	-
5-alpha-pregnan-3beta,20alpha-disulfate	MR-PRESSO	15	-0.86	0.31	0.0160	0.42	0.23	0.78	>0.05
Gamma-glutamylmethionine	Inverse variance weighted	9	2.03	1.00	0.0420	7.61	1.07	53.99	-
Gamma-glutamylmethionine	Weighted median	9	1.17	1.14	0.3050	3.23	0.34	30.34	-
Gamma-glutamylmethionine	MR-PRESSO	9	2.03	1.00	0.0770	7.61	1.07	54.05	>0.05
Valerate	Inverse variance weighted	10	-4.97	1.30	0.0000	0.01	0.00	0.09	-
Valerate	Weighted median	10	-5.41	1.77	0.0020	0.00	0.00	0.14	-
Valerate	MR-PRESSO	10	-4.97	1.30	0.0040	0.01	0.00	0.09	>0.05
Isovalerylcarnitine	Inverse variance weighted	21	1.39	0.66	0.0340	4.01	1.11	14.53	-
Isovalerylcarnitine	Weighted median	21	1.20	0.94	0.2020	3.34	0.52	21.24	-
Isovalerylcarnitine	MR-PRESSO	21	1.33	0.54	0.0220	3.78	1.32	10.84	>0.05

1-arachidonoylglycerophosphoethanolamine	Inverse variance weighted	26	-1.65	0.81	0.0430	0.19	0.04	0.95	-
1-arachidonoylglycerophosphoethanolamine	Weighted median	26	-1.63	1.16	0.1600	0.20	0.02	1.91	-
1-arachidonoylglycerophosphoethanolamine	MR-PRESSO	26	-1.58	0.67	0.0260	0.21	0.06	0.77	>0.05
Hydroxyisovaleroyl carnitine	Inverse variance weighted	8	2.39	0.97	0.0130	10.96	1.65	72.86	-
Hydroxyisovaleroyl carnitine	Weighted median	8	1.63	1.17	0.1620	5.11	0.52	50.44	-
Hydroxyisovaleroyl carnitine	MR-PRESSO	8	2.20	0.93	0.0450	9.00	1.46	55.47	>0.05

Table S4. Sensitive results of the associations between serum metabolites and appendicular lean mass as well as the global test of MR-PRESSO.

Metabolites	Method	N SNP	Beta	se	P	lo_ci	up_ci	P global test
Mannose	Inverse variance weighted	21	0.91	0.29	0.0020	0.34	1.48	-
	Weighted median	21	0.72	0.26	0.0060	0.20	1.23	-
	MR-PRESSO	21	0.91	0.29	0.0050	0.34	1.48	<0.05
Citrate	Inverse variance weighted	46	0.46	0.19	0.0130	0.10	0.83	-
	Weighted median	46	0.15	0.21	0.4570	-0.25	0.56	-
	MR-PRESSO	46	0.48	0.18	0.0100	0.13	0.83	<0.05
Guanosine	Inverse variance weighted	13	-0.12	0.06	0.0500	-0.24	0.00	
	Weighted median	13	-0.13	0.08	0.1180	-0.29	0.03	-
	MR-PRESSO	13	-0.12	0.05	0.0400	-0.22	-0.02	>0.05
Betaine	Inverse variance weighted	25	-0.89	0.31	0.0040	-1.50	-0.28	-
	Weighted median	25	-0.19	0.21	0.3670	-0.61	0.23	-
	MR-PRESSO	25	-0.87	0.30	0.0090	-1.47	-0.27	<0.05
Beta-hydroxyisovalerate	Inverse variance weighted	23	-0.49	0.20	0.0140	-0.89	-0.10	-
	Weighted median	23	-0.03	0.19	0.8800	-0.41	0.35	-
	MR-PRESSO	23	-0.47	0.20	0.0250	-0.86	-0.09	<0.05
3-methylhistidine	Inverse variance weighted	11	-0.14	0.06	0.0160	-0.25	-0.03	-
	Weighted median	11	-0.01	0.08	0.9350	-0.16	0.15	-
	MR-PRESSO	11	-0.14	0.05	0.0370	-0.24	-0.04	>0.05
Serine	Inverse variance weighted	35	0.91	0.42	0.0320	0.08	1.74	-
	Weighted median	35	0.40	0.23	0.0860	-0.06	0.86	-
	MR-PRESSO	35	0.90	0.41	0.0360	0.09	1.72	<0.05
Hexanoylcarnitine	Inverse variance weighted	19	-0.40	0.16	0.0160	-0.72	-0.07	-
	Weighted median	19	-0.26	0.11	0.0150	-0.48	-0.05	-

	MR-PRESSO	19	-0.40	0.16	0.0270	-0.72	-0.07	<0.05
Glycine	Inverse variance weighted	26	0.51	0.16	0.0020	0.19	0.84	-
	Weighted median	26	0.99	0.12	0.0000	0.75	1.23	-
	MR-PRESSO	26	0.51	0.16	0.0050	0.19	0.84	<0.05
O-methylascorbate	Inverse variance weighted	49	-0.44	0.15	0.0030	-0.74	-0.15	-
	Weighted median	49	-0.66	0.15	0.0000	-0.95	-0.37	-
	MR-PRESSO	49	-0.44	0.15	0.0050	-0.74	-0.15	<0.05
Docosapentaenoate	Inverse variance weighted	11	0.45	0.19	0.0160	0.08	0.81	-
	Weighted median	11	0.39	0.18	0.0280	0.04	0.74	-
	MR-PRESSO	11	0.44	0.17	0.0260	0.11	0.78	<0.05
1-arachidonoylglycerophosphocholine	Inverse variance weighted	21	0.33	0.12	0.0080	0.08	0.57	-
	Weighted median	21	0.43	0.14	0.0020	0.17	0.70	-
	MR-PRESSO	21	0.33	0.12	0.0160	0.08	0.57	-
Decanoylcarnitine	Inverse variance weighted	15	-0.34	0.11	0.0020	-0.55	-0.12	-
	Weighted median	15	-0.23	0.10	0.0240	-0.42	-0.03	-
	MR-PRESSO	15	-0.34	0.11	0.0080	-0.55	-0.12	<0.05
1-palmitoyl glycerophosphocholine	Inverse variance weighted	34	-0.48	0.23	0.0380	-0.92	-0.03	-
	Weighted median	34	-0.13	0.26	0.6180	-0.63	0.38	-
	MR-PRESSO	34	-0.48	0.23	0.0460	-0.92	-0.03	<0.05
1-oleoylglycerophosphocholine	Inverse variance weighted	17	-0.37	0.14	0.0100	-0.65	-0.09	-
	Weighted median	17	-0.26	0.20	0.1940	-0.66	0.13	-
	MR-PRESSO	17	-0.37	0.14	0.0170	-0.64	-0.10	>0.05
10-heptadecenoate (17:1n7)	Inverse variance weighted	6	0.40	0.19	0.0340	0.03	0.78	-
	Weighted median	6	0.40	0.25	0.1030	-0.08	0.88	-
	MR-PRESSO	6	0.40	0.11	0.0150	0.19	0.62	>0.05
Isovalerylcarnitine	Inverse variance weighted	21	-0.45	0.18	0.0150	-0.81	-0.09	-

	Weighted median	21	0.00	0.15	0.9780	-0.29	0.28	-
	MR-PRESSO	21	-0.44	0.18	0.0230	-0.79	-0.09	<0.05
Nonanoylcarnitin	Inverse variance weighted	16	0.13	0.07	0.0480	0.00	0.26	-
	Weighted median	16	0.11	0.07	0.1320	-0.03	0.25	-
	MR-PRESSO	16	0.13	0.07	0.0670	0.00	0.26	>0.05
Tetradecanedioate	Inverse variance weighted	20	0.13	0.06	0.0260	0.02	0.25	-
	Weighted median	20	0.08	0.08	0.3120	-0.08	0.24	-
	MR-PRESSO	20	0.14	0.06	0.0270	0.03	0.26	>0.05
Octadecanedioate	Inverse variance weighted	10	0.32	0.16	0.0430	0.01	0.63	-
	Weighted median	10	0.32	0.17	0.0610	-0.01	0.66	-
	MR-PRESSO	10	0.32	0.16	0.0740	0.01	0.63	<0.05
5α-Adiol disulfate	Inverse variance weighted	18	0.14	0.07	0.0280	0.02	0.27	-
	Weighted median	18	0.11	0.06	0.0470	0.00	0.22	-
	MR-PRESSO	18	0.14	0.07	0.0420	0.02	0.27	<0.05
4α-Adiol disulfate 2	Inverse variance weighted	19	0.33	0.14	0.0180	0.06	0.61	-
	Weighted median	19	0.11	0.12	0.3750	-0.13	0.35	-
	MR-PRESSO	19	0.33	0.14	0.0300	0.06	0.61	<0.05

Table S5. Sensitive results of the associations between serum metabolites and grip strength as well as the global test of MR-PRESSO.

Metabolites	Method	N SNP	Beta	se	P	lo_ci	up_ci	P global test
Laurate (12:0)	Inverse variance weighted	41	-1.04	0.42	0.0137	-1.86	-0.21	-
	Weighted median	41	-0.65	0.47	0.1667	-1.56	0.27	-
	MR-PRESSO	41	-1.02	0.41	0.0169	-1.83	-0.22	<0.05
Gamma-glutamyltyrosine	Inverse variance weighted	47	0.76	0.38	0.0479	0.01	1.51	-
	Weighted median	47	0.48	0.46	0.3062	-0.44	1.39	-
	MR-PRESSO	47	0.75	0.38	0.0516	0.01	1.49	<0.05
Glucose	Inverse variance weighted	38	-0.80	0.40	0.0465	-1.59	-0.01	>0.05
	Weighted median	38	-0.50	0.58	0.3899	-1.64	0.64	-
	MR-PRESSO	38	-0.84	0.39	0.0389	-1.62	-0.07	<0.05
1-oleoylglycerol (1-monoolein)	Inverse variance weighted	16	-0.54	0.26	0.0404	-1.05	-0.02	-
	Weighted median	16	-0.52	0.28	0.0652	-1.07	0.03	-
	MR-PRESSO	16	-0.54	0.26	0.0583	-1.05	-0.02	<0.05
Hyodeoxycholate	Inverse variance weighted	17	0.29	0.12	0.0170	0.05	0.52	>0.05
	Weighted median	17	0.33	0.15	0.0305	0.03	0.62	-
	MR-PRESSO	17	0.28	0.11	0.0235	0.06	0.50	<0.05
Androsterone sulfate	Inverse variance weighted	22	-0.17	0.07	0.0142	-0.30	-0.03	>0.05
	Weighted median	22	-0.25	0.09	0.0043	-0.42	-0.08	-
	MR-PRESSO	22	-0.17	0.07	0.0230	-0.30	-0.03	<0.05
Glycine	Inverse variance weighted	26	0.66	0.24	0.0059	0.19	1.13	>0.05
	Weighted median	26	1.01	0.26	0.0001	0.51	1.52	-
	MR-PRESSO	26	0.66	0.24	0.0108	0.19	1.13	<0.05
3-dehydrocarnitine	Inverse variance weighted	27	-1.51	0.39	0.0001	-2.27	-0.75	-
	Weighted median	27	-1.14	0.43	0.0086	-1.99	-0.29	-
	MR-PRESSO	27	-1.51	0.39	0.0006	-2.27	-0.75	<0.05

1-heptadecanoyl glycerophosphocholine	Inverse variance weighted	10	1.10	0.42	0.0098	0.26	1.93	-
	Weighted median	10	0.67	0.45	0.1369	-0.21	1.55	-
	MR-PRESSO	10	1.10	0.42	0.0296	0.26	1.93	<0.05
Epiandrosterone sulfate	Inverse variance weighted	13	-0.33	0.10	0.0015	-0.53	-0.13	>0.05
	Weighted median	13	-0.44	0.13	0.0008	-0.70	-0.18	-
	MR-PRESSO	13	-0.33	0.10	0.0079	-0.53	-0.13	<0.05
Isovalerylcarnitine	Inverse variance weighted	21	-1.51	0.41	0.0002	-2.31	-0.71	-
	Weighted median	21	-0.69	0.36	0.0561	-1.41	0.02	-
	MR-PRESSO	21	-1.51	0.40	0.0010	-2.28	-0.73	<0.05

Table S6. Detailed information on selected single nucleotide polymorphisms robustly and independently related to sarcopenic indices.

Trait	N SNP	Variance	Minimum F statistic
Appendicular lean mass	436	9.5%	28.841
Grip strength	135	1.4%	29.897

Instrumental variables were significantly associated with appendicular lean mass and grip strength ($P<5\times10^{-8}$) and were independent (linkage disequilibrium r2<0.001 within 10,000 kb).

Table S7. Results of the reverse associations between sarcopenic indices and identified metabolites.

Metabolites	Exposure	Beta	se	P	lo_ci	up_ci
4-acetamidobutanoate	Appendicular lean mass	0.002	0.005	0.728	-0.008	0.012
	Grip strength	-0.039	0.017	0.018	-0.072	-0.006
Biliverdin	Appendicular lean mass	0.007	0.011	0.509	-0.015	0.029
	Grip strength	-0.039	0.033	0.233	-0.104	0.026
Taurocholate	Appendicular lean mass	-0.020	0.023	0.373	-0.065	0.025
	Grip strength	-0.038	0.072	0.594	-0.179	0.103
Myo-inositol	Appendicular lean mass	-0.000	0.005	0.959	-0.010	0.010
	Grip strength	-0.013	0.016	0.435	-0.044	0.018
Phenyllactate	Appendicular lean mass	0.006	0.007	0.385	-0.008	0.020
	Grip strength	0.001	0.023	0.962	-0.044	0.046
Levulinate (4-oxovalerate)	Appendicular lean mass	-0.007	0.006	0.256	-0.019	0.005
	Grip strength	-0.051	0.018	0.004	-0.086	-0.016
Acetylcarnitine	Appendicular lean mass	-0.009	0.006	0.189	-0.021	0.003
	Grip strength	0.003	0.021	0.872	-0.038	0.044
Docosapentaenoate	Appendicular lean mass	-0.011	0.011	0.279	-0.033	0.011
	Grip strength	-0.013	0.032	0.693	-0.076	0.050
5-alpha-pregnan-3beta,20alpha-disulfate	Appendicular lean mass	-0.010	0.024	0.673	-0.057	0.037
	Grip strength	-0.248	0.076	0.001	-0.397	-0.099
Gamma-glutamylmethionine	Appendicular lean mass	-0.013	0.011	0.220	-0.035	0.009
	Grip strength	-0.057	0.037	0.118	-0.130	0.016
Valerate	Appendicular lean mass	-0.001	0.009	0.879	-0.019	0.017
	Grip strength	-0.034	0.028	0.217	-0.089	0.021
Isovalerylcarnitine	Appendicular lean mass	-0.007	0.010	0.498	-0.027	0.013
	Grip strength	-0.034	0.031	0.273	-0.095	0.027

1-arachidonoylglycerophosphoethanolamine	Appendicular lean mass	0.009	0.007	0.188	-0.005	0.023
	Grip strength	0.004	0.024	0.871	-0.043	0.051
Hydroxyisovaleroyl carnitine	Appendicular lean mass	-0.014	0.010	0.161	-0.034	0.006
	Grip strength	-0.020	0.034	0.565	-0.087	0.047
Mannose	Appendicular lean mass	0.008	0.008	0.319	-0.008	0.024
	Grip strength	-0.037	0.021	0.073	-0.078	0.004
Citrate	Appendicular lean mass	-0.004	0.006	0.482	-0.016	0.008
	Grip strength	0.022	0.018	0.232	-0.013	0.057
Guanosine	Appendicular lean mass	0.006	0.019	0.734	-0.031	0.043
	Grip strength	0.040	0.062	0.519	-0.082	0.162
Betaine	Appendicular lean mass	-0.005	0.006	0.442	-0.017	0.007
	Grip strength	-0.013	0.023	0.563	-0.058	0.032
Beta-hydroxyisovalerate	Appendicular lean mass	-0.014	0.008	0.057	-0.030	0.002
	Grip strength	-0.024	0.025	0.327	-0.073	0.025
3-methylhistidine	Appendicular lean mass	-0.018	0.023	0.451	-0.063	0.027
	Grip strength	-0.024	0.074	0.742	-0.169	0.121
Serine	Appendicular lean mass	-0.013	0.007	0.052	-0.027	0.001
	Grip strength	-0.011	0.023	0.645	-0.056	0.034
Hexanoylcarnitine	Appendicular lean mass	-0.014	0.009	0.113	-0.032	0.004
	Grip strength	-0.002	0.030	0.934	-0.061	0.057
Glycine	Appendicular lean mass	0.012	0.011	0.272	-0.010	0.034
	Grip strength	0.053	0.053	0.319	-0.051	0.157
O-methylascorbate	Appendicular lean mass	-0.003	0.006	0.569	-0.015	0.009
	Grip strength	-0.026	0.017	0.129	-0.059	0.007
1-arachidonoylglycerophosphocholine	Appendicular lean mass	0.000	0.008	0.993	-0.016	0.016
	Grip strength	0.014	0.027	0.609	-0.039	0.067

Decanoylcarnitine	Appendicular lean mass	-0.010	0.011	0.365	-0.032	0.012
	Grip strength	0.0308	0.036	0.390	-0.040	0.101
1-palmitoyl glycerophosphocholine	Appendicular lean mass	-0.004	0.005	0.400	-0.014	0.006
	Grip strength	-0.000	0.017	0.980	-0.033	0.033
1-oleoylglycerophosphocholine	Appendicular lean mass	0.004	0.007	0.636	-0.010	0.018
	Grip strength	0.012	0.025	0.634	-0.037	0.061
10-heptadecenoate (17:1n7)	Appendicular lean mass	-0.010	0.009	0.298	-0.028	0.008
	Grip strength	-0.022	0.028	0.437	-0.077	0.033
Nonanoylcarnitin	Appendicular lean mass	0.007	0.013	0.564	-0.018	0.032
	Grip strength	-0.010	0.040	0.807	-0.088	0.068
Tetradecanedioate	Appendicular lean mass	0.000	0.013	0.994	-0.025	0.025
	Grip strength	0.034	0.044	0.431	-0.052	0.120
Octadecanedioate	Appendicular lean mass	-0.005	0.010	0.612	-0.025	0.015
	Grip strength	0.028	0.031	0.364	-0.033	0.089
5α-Adiol disulfate	Appendicular lean mass	0.011	0.018	0.551	-0.024	0.046
	Grip strength	-0.056	0.060	0.352	-0.174	0.062
4α-Adiol disulfate 2	Appendicular lean mass	0.008	0.012	0.504	-0.016	0.032
	Grip strength	0.000	0.039	0.990	-0.076	0.076
Laurate (12:0)	Appendicular lean mass	-0.005	0.005	0.357	-0.015	0.005
	Grip strength	-0.024	0.017	0.165	-0.057	0.009
Gamma-glutamyltyrosine	Appendicular lean mass	0.007	0.005	0.166	-0.003	0.017
	Grip strength	0.005	0.016	0.771	-0.026	0.036
Glucose	Appendicular lean mass	0.002	0.004	0.609	-0.006	0.010
	Grip strength	-0.025	0.014	0.070	-0.052	0.002
1-oleoylglycerol (1-monoolein)	Appendicular lean mass	-0.002	0.015	0.922	-0.031	0.027
	Grip strength	0.022	0.048	0.638	-0.072	0.116

Hyodeoxycholate	Appendicular lean mass	-0.008	0.019	0.665	-0.045	0.029
	Grip strength	0.024	0.059	0.687	-0.092	0.140
Androsterone sulfate	Appendicular lean mass	0.011	0.019	0.562	-0.026	0.048
	Grip strength	-0.017	0.059	0.776	-0.133	0.099
3-dehydrocarnitine	Appendicular lean mass	-0.009	0.007	0.172	-0.023	0.005
	Grip strength	-0.036	0.021	0.085	-0.077	0.005
1-heptadecanoyl glycerophosphocholine	Appendicular lean mass	-0.002	0.010	0.882	-0.022	0.018
	Grip strength	0.000	0.033	0.995	-0.065	0.065
Epiandrosterone sulfate	Appendicular lean mass	0.001	0.017	0.932	-0.032	0.034
	Grip strength	-0.006	0.054	0.909	-0.112	0.100

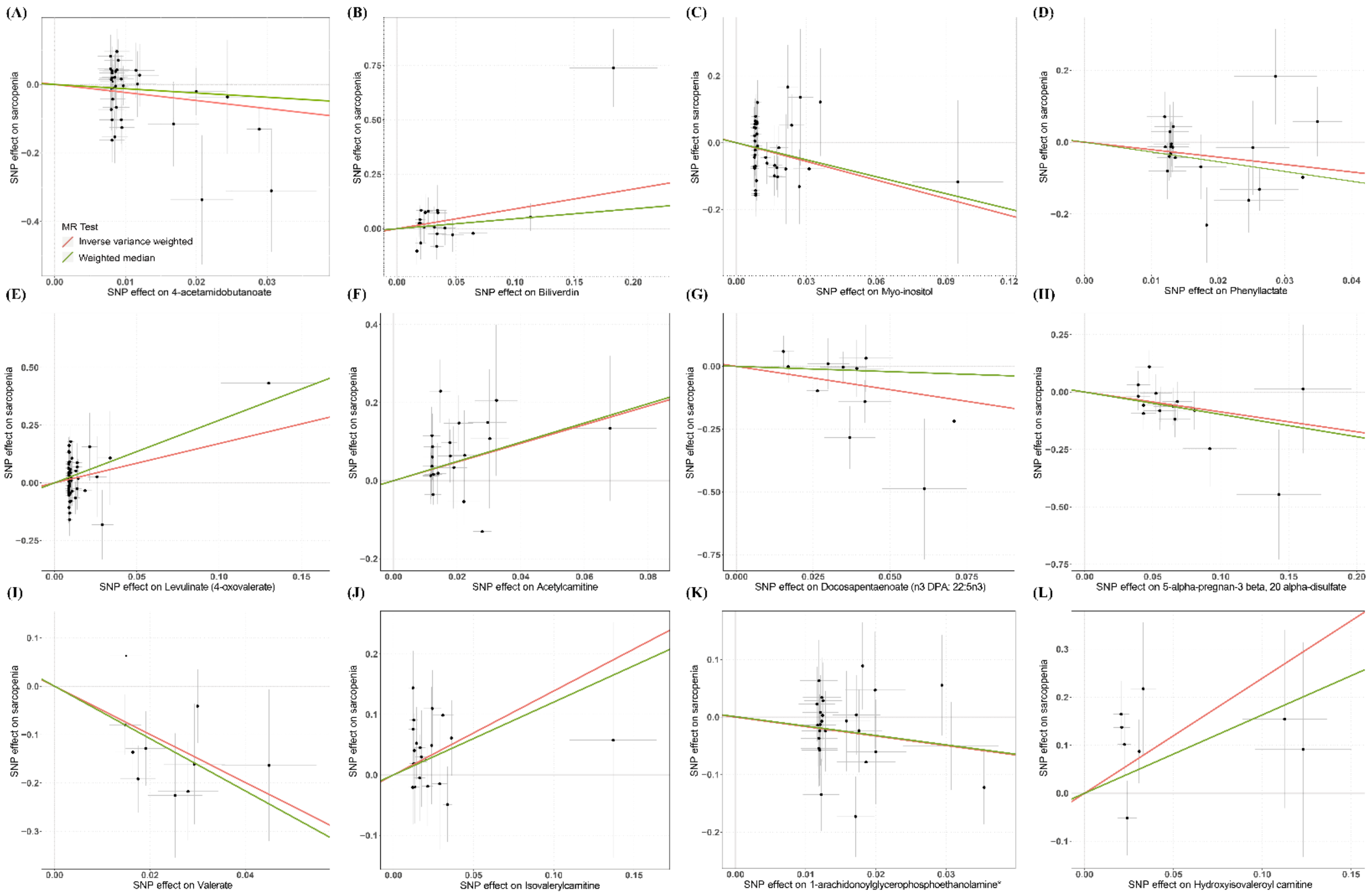

Figure S1. Suggestive significant association between serum metabolites with sarcopenia. In A-L, red lines represent estimations with IVW method and green lines represent estimations with weighted median method. Scatter plot for causal effect of (A) 4-acetamidobutanoate genus,

(B) Biliverdin genus, (C) Myo-inositol genus, (D) Phenyllactate genus, (E) Levulinate (4-oxovalerate) genus, (F) Acetylcarnitine genus, (G) Docosapentaenoate genus, (H) 5-alpha-pregnan-3beta,20alpha-disulfate genus, (I) Valerate genus, (J) Isovalerylcarnitine genus, (K) 1-arachidonoylglycerophosphoethanolamine genus, (L) Hydroxyisovaleroyl carnitine genus on sarcopenia. SNPs, single nucleotide polymorphisms; IVW, inverse-variance weighted; MR, Mendelian randomization.
